# Genome Sequence of a Unique *Magnaporthe oryzae* RMg-Dl Isolate from India That Causes Blast Disease in Diverse Cereal Crops, Obtained Using PacBio Single-Molecule and Illumina HiSeq2500 Sequencing

**DOI:** 10.1128/genomeA.01570-16

**Published:** 2017-02-16

**Authors:** Aundy Kumar, Neelam Sheoran, Ganesan Prakash, Arpita Ghosh, Surendra K. Chikara, Hosahatti Rajashekara, Uday Dhari Singh, Rashmi Aggarwal, Rakesh Kumar Jain

**Affiliations:** a Division of Plant Pathology, ICAR-Indian Agricultural Research Institute, New Delhi, India; b Eurofins Genomics India Private Limited, Bengaluru, Karnataka, India

## Abstract

The whole-genome assembly of a unique rice isolate from India, *Magnaporthe oryzae* RMg-Dl that causes blast disease in diverse cereal crops is presented. Analysis of the 34.82 Mb genome sequence will aid in better understanding the genetic determinants of host range, host jump, survival, pathogenicity, and virulence factors of *M. oryzae*.

## GENOME ANNOUNCEMENT

In India, the blast disease caused by *Magnaporthe oryzae* (Hebert) Barr (anamorph: *Pyricularia grisea*) is one of the major production constraints in both aromatic and nonaromatic rice (1). Apart from rice, natural infection of *M. oryzae* is recorded in finger millet and pearl millet in India (2, 3). The pathogen is also a major threat to other important cereals including wheat, barley, and rye (4, 5). In recent years, *M. oryzae* has become a model organism to study plant-pathogen interaction (6, 7). The *M. oryzae* RMg-Dl isolate studied here originated from the blast infected rice cultivar Swarna in the Madhubani region in the Bihar state of India. The isolate RMg-Dl was able to knock down several blast resistance (*R*) genes except *Pi54* and Tetep (*Pi-1*, *Pi-kh*, *Pi-ta*, and* Pi-ta^2^+*). Interestingly, the isolate was highly pathogenic on other cereals including wheat, oat, and barley under experimental conditions but not on finger millet and pearl millet. The monosporidial culture of the RMg-Dl isolate maintained on rice straw extract oat agar medium (8) was used for extracting high-quality genomic DNA. The quality and quantity of DNA was assessed on 0.8% agarose gel, Nanodrop 2000 (A260/280), as well as the Qubit 3.0 Fluorometer (Thermo Scientific Inc, USA).

Prior to whole-genome sequencing, the genomic DNA identity was confirmed as *M. oryzae* by sequencing a partial 18S rDNA gene on ABI3730xl. The paired-end (PE) sequencing library was prepared using the Illumina TruSeq Nano DNA HT library preparation kit. For ultralong read sequencing on the PacBio platform, high molecular weight DNA was used to prepare a single-molecule real-time (SMRT)-bell library of size 5 to 8 kb. The amplified library was analyzed in Bioanalyzer-2100 (Agilent Technologies, USA) using a high sensitivity DNAchip as per the manufacturer’s instructions. While the PE Illumina library was sequenced using 2 × 125 bp chemistry in HiSeq-2500, the SMRT bell library was sequenced on PacBio-RSII using P6-C4 chemistry. Whereas a total of 16.3 Gb of data representing high-quality reads were generated on HiSeq 2500, the PacBio-RSII platform yielded 1.5 Gb of data. High-quality paired-end short reads of Illumina HiSeq-2500 and long reads of PacBio-RSII were *de nova* assembled using a hybrid approach by SPAdes (version 1.5.2) with default parameters (9).

In total, the assembly of 996 scaffolds resulted in a genome size of 34.82 Mb with an *N*_50_ value of 45,894 bp. The gene prediction was performed with the help of Augustus (version 3.2.1) using *Saccharomyces cerevisiae* S288C as a model (10). A total of 12,747 genes in the range of 201 to 14,916 bp were predicted with an average size of 1,007 bp. Predicted genes were mapped on reference canonical pathways and classified using an automated KASS server in KEGG (http://www.genome.jp/kaas-bin/kaas_main). Functional annotation of the genes was performed using the BLASTx program. Genes governing pathogenesis, virulence effectors, and host range were annotated using the PHIbase V-4.1 database or literature (11, 12). In addition, a total of 300 transposons were identified using Transposons PSI in *M. oryzae* (http://transposonpsi.sf.net). The information presented here will enable further study of the genetic and functional characteristics of rice-infecting broad host range *M. oryzae* RMg-Dl.

### Accession number(s).

This whole-genome shotgun project has been deposited at DDBJ/ENA/GenBank under the accession number MBSD00000000. The version described in this paper is version MBSD02000000.
